# Supplementary material for: IL-2Rα KO mice exhibit maternal microchimerism and reveal nuclear localization of IL-2Rα in lymphoid and non-lymphoid cells
Source: Front Immunol. 2024 May 15;15:1369818. doi: 10.3389/fimmu.2024.1369818 (PMC11133634; doi:10.3389/fimmu.2024.1369818)
Supplement: Supplementary file 1 [file DataSheet_1.pdf]

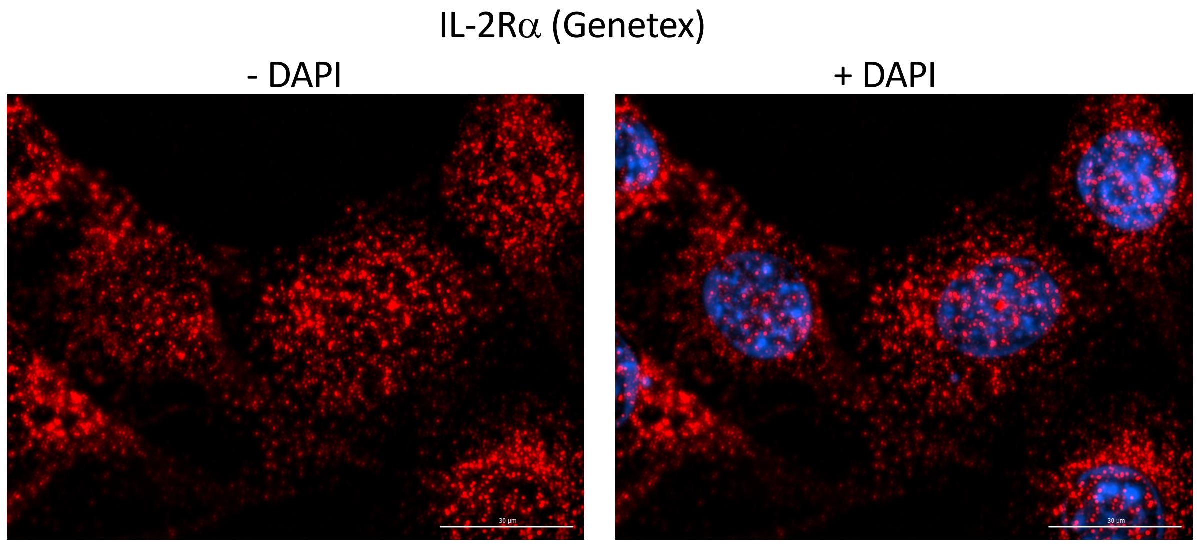

Supplemental Figure 1. Mouse anti-IL-2Rα, clone 1B5D12, detects IL-2Rα in both the nucleus and cytoplasm/membrane. Mouse VSMC were grown to approximately 70% confluence, then fixed and stained using a mouse monoclonal anti-IL-2Rα antibody, clone 1B5D12. Images were captured as a Z stack with a Cytation multi-mode imaging plate reader using a 1.6 μ depth of field. The image shown is 1.6 μ into the stack. Scale bar = 30 μ.

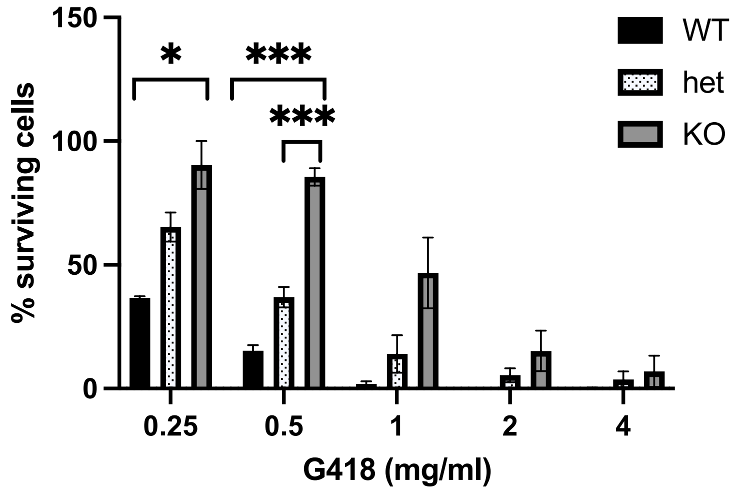

Supplemental Figure 2. Sensitivity of WT, heterozygous, and IL-2Rα KO VSMC to G418. VSMC, isolated from mice with genotypes as indicated, were cultured with increasing concentrations of G418 for 96h. Remaining cells were counted using a nuclear stain. Survival is expressed as a percentage of remaining cells using cells cultured without G418 as the denominator. Results shown represent the average ± SEM of three experiments.

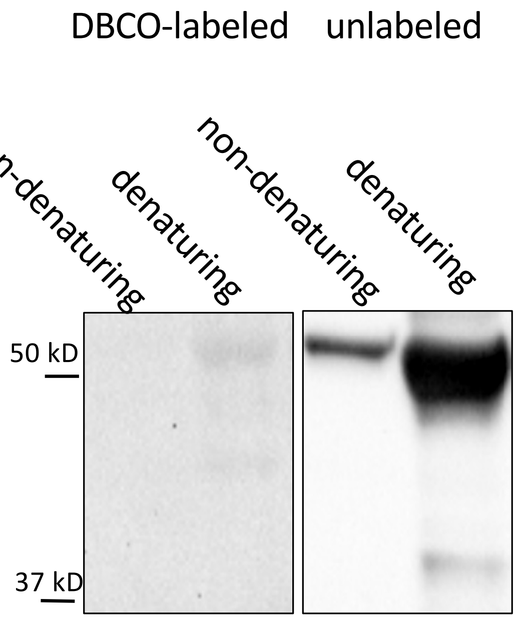

Supplemental Figure 3. DBCO label interferes with detection of IL-2Rα by Western blot. IL-2Rα was isolated from nuclear fractions of VSMC lysates by immunoprecipitation using a rabbit polyclonal anti-IL-2Rα from BosterBio. IL-2Rα protein was eluted from beads (directly conjugated with antibody) using either a non-denaturing or denaturing buffer. DBCO was added to a portion of the lysates as indicated. Lysates were subjected to Western blot analysis and probed with anti-phospho IL-2Rα (ser268). Similar issues with detection were noted when probing with rabbit anti-IL-2Rα polyclonal (BosterBio) or mouse anti-IL-2Rα monoclonal (clone 1B5D12, Genetex) antibodies.
